# Therapeutic potential of cannabinoids for treating atopic dermatitis

**DOI:** 10.1186/s42238-025-00317-4

**Published:** 2025-08-16

**Authors:** Adriel Aparecido Geraldo Stoco, Priscila Gava Mazzola

**Affiliations:** Faculty of Pharmaceutical Sciences, Universidade Estadual de Campinas, Rua Cândido Portinari, 200, Cidade Universitária Zeferino Vaz, Campinas, São Paulo 13083—871 Brazil

**Keywords:** Atopic dermatitis, Cannabinoids, Cannabidiol, Δ-9-tetrahydrocannabinol, Palmitoylethanolamide, Dronabinol

## Abstract

*This review aims to assess the therapeutic potential of cannabinoids as complementary treatments for atopic dermatitis.* Atopic dermatitis (AD) is a skin disease characterized by the loss of skin barrier function that promotes subsequent symptoms such as intense itching, xerosis and inflammation. Several treatments are available, particularly topical approaches, which are crucial for both acute and chronic management of the disease. The main objectives of topical treatments are to promote skin hydration and reduce itching and immune responses, typically through lotions and topical medications such as glucocorticoids. However, the long-term use of glucocorticoids presents certain disadvantages, highlighting the need for new therapeutic options to minimize adverse effects and providing a broader range of choices for both physicians and patients to find the best alternative for each case. Research involving cannabinoids, which can be endogenous, plant-based or synthetic, has intensified in recent years to evaluate the therapeutic potential of these compounds for skin conditions, including AD. Studies suggest that phytocannabinoids such as cannabidiol (CBD) and Δ-9-tetrahydrocannabinol (THC), along with endogenous and synthetic compounds such as palmitoyletanolamide (PEA) and dronabinol, can improve AD symptoms, primarily because of their anti-inflammatory, antipruritic and antioxidant properties. Additionally, some cannabinoids exhibit antimicrobial effects. Despite these promising results, the use of cannabinoids in AD treatment requires further investigation to better understand their efficiency and safety, necessitating high-accuracy clinical and preclinical trials.

## Introduction

Atopic dermatitis (AD) is a multifactorial disease that affects thousands of people around the world and significantly impacts the quality of life of many patients, including young people and adolescents (Fotopoulou et al. 2018; Tackett et al. 2020).

Various drug treatment options are available, divided between topical and systemic approaches, which, despite their indispensable importance, do not always improve the symptoms of the disease, especially constant pruritus (Liu et al. 2024). Pruritus is a medical term for itching, and it is defined as an unpleasant cutaneous sensation leading to the desire to scratch (Bernhard 2005).

Topical treatments are indispensable for controlling this disease, and the use of moisturizers and corticosteroids are the main representatives of this therapeutic category (Chu et al. 2024). Moisturizers vary in their composition and the benefits they can offer to the skin of patients with AD and therefore differ considerably in terms of the cost of their acquisition, whereas topical corticosteroids (TCSs), despite their undeniable contribution to symptom remission, have a limited time of use owing to the undesirable side effects that prolonged use can offer, such as atrophy, striae, rosacea, perioral dermatitis, acne and purpura (Hengge et al. 2006). In this sense, additional and alternative topical approaches can help overcome the deficiencies of traditional treatments, and the application of plant, synthetic or endogenous cannabinoids in topical formulations may be promising as an adjuvant treatment for this disease (Morin et al. 2021).

The topical use of cannabinoids for treating skin conditions remains limited due to insufficient clinical experience and a lack of robust evidence regarding their efficacy and safety (Souza et al. 2022). References concerning adverse effects associated with the topical use of cannabinoids are limited, with only a few well-documented case reports available, most of which are related to occupational or recreational exposure. These findings highlight the need for safety research specifically focused on cannabinoids intended for exclusive topical use (Souza et al. 2022; Heyne et al. 2024).

In addition, there is great divergence between legislation around the world on the use of cannabinoid derivatives in medicine, with some countries adopting this type of therapy to some degree, such as Canada, which in October 2018, authorized the patients by their health care providers to access cannabis for medical purposes, and others with more restrictive policies, such as South Korea, where there is a strict narcotics control law that prohibits and penalizes practically all forms of consumption (Cannabis for medical purposes under the Cannabis Act: information and improvements - Canada.ca. 2025; Is cannabis legal in South Korea - 2025). These differences and legislative barriers make it difficult to conduct research and apply scientific methods that generate reliable and secure data on the subject (Wilson et al. 2017).

Although there is a lack of based evidence available on clinical trials methods, there are some indications that the use of cannabinoids can have a significantly positive effect on various skin conditions, including atopic dermatitis (Gao et al. 2022; Maghfour et al. 2020). Preclinical studies have demonstrated the therapeutic potential of cannabinoids administered via both topical and oral routes. Topical formulations incorporating N-palmitoylethanolamide (PEA), an endocannabinoid analogue, and cannabidiol (CBD) have shown promise, as well as oral administration of dronabinol, a synthetic analogue of Δ^9^-tetrahydrocannabinol (Δ^9^-THC) (Eberlein et al. 2008; Gao et al. 2022; Morin et al. 2021). The applications of these compounds as therapeutic potential in the treatment of atopic dermatitis, as well as other dermatological diseases, extend beyond the vast phytochemical repertoire offered by *Cannabis sativa*, representing a source of knowledge to be explored, with marked contributions to pharmaceutical and agricultural technological development.

This review aims to assess the therapeutic potential of cannabinoids in the treatment of atopic dermatitis, with a focus on clinical and preclinical evidence. This review explores how cannabinoids, including natural, synthetic, and endocannabinoid compounds, can be used in topical formulations to alleviate symptoms of AD. Additionally, the review highlights the challenges related to limited research, legislative barriers, and the need for further studies to establish the safety and efficacy of cannabinoid-based treatments for atopic dermatitis.

## Methods

The most relevant articles regarding atopic dermatitis and cannabinoids with therapeutic potential for the treatment of atopic dermatitis were selected from 2020–2024 from the Science Direct, Pubmed and Web of Science databases. The terms used in the data search were “atopic dermatitis”, “eczema”, “cannabinoids”, “cannabis sativa”, “palmitoylethanolamide”, “Δ−9-tetrahydrocannabinol”, “cannabidiol”, “dronabinol”, “Itching”, “scratching”, “filaggrin”, “glucocorticoids” and “topical medications”. References older than 2020 were included to describe methods and concepts.

## Atopic dermatitis

Atopic dermatitis (AD), one of several conditions known as eczema, is a chronic inflammatory skin disease characterized by intense itching and dry and often inflamed skin (Weidinger et al. 2018). The onset of the disease usually occurs between 3 and 6 months of age, with up to 60% of patients developing the disease in the first year of life and 80% to 90% by the first 5 years of age (Torres et al. 2019).

Approximately 204 million people worldwide are affected by AD, with considerable variation across age groups and geographic regions (Weidinger and Novak 2016). In children and adolescents, prevalence is influenced by socioeconomic factors, and the condition is frequently associated with other atopic comorbidities (Laughter et al. 2021). In adults, available data are limited and often underestimated, with a higher prevalence observed among females (Odhiambo et al. 2009).

AD pathogenesis is multifactorial, involving genetic predisposition, environmental exposures (e.g., pollutants, mold, tobacco smoke, biocides), and the activation of type 2 immune responses. Immune activation occurs even in non-lesional skin and becomes more pronounced in acute and chronic lesions. Additionally, interactions between cytokines and peripheral neurons contribute to the intense pruritus that characterizes AD (Guttman-Yassky et al. 2025). The skin’s barrier function is impaired in AD due to both structural and immunological dysfunctions, particularly loss-of-function mutations in the filaggrin (FLG) gene (Jungersted et al. 2010).

The onset of AD can significantly impact a patient’s quality of life from childhood through adulthood, with notable effects on academic, social, and professional performance (Fotopoulou et al. 2018; Tackett et al. 2020; Torres et al. 2019).

### Pathophysiology

The skin, the largest organ of the human body, functions as a critical protective barrier against external agents such as extreme temperatures, mechanical trauma, microorganisms, and toxins. Furthermore, it plays a fundamental role in preventing the loss of essential substances necessary for physiological homeostasis (Proksch et al. 2008). Structurally complex, the skin is composed of three functionally interdependent layers: the epidermis, dermis, and hypodermis, arranged from the outermost to the innermost layers, respectively (Sullivan and Myers 2021). In individuals affected by atopic dermatitis (AD), these structures are disrupted and, consequently, more susceptible to environmental insults, frequently triggering type 2 immune responses (Lyubchenko et al. 2021).

Growing evidence suggests that maternal exposure to environmental pollutants such as heavy metals and fluoride during pregnancy is associated with an increased risk of allergic diseases, including atopic dermatitis and food allergies in early childhood (Kampouri et al. 2023). Domestic environmental characteristics, including housing size, shared bedrooms, mold and dampness, cigarette smoke exposure, among others, have been associated with a higher prevalence of AD. In contrast, rural areas or homes with greater natural ventilation tend to show lower incidence rates (Zhang et al. 2023). Early exposure to dust mites and household dust has also been correlated with increased incidence of asthma, rhinitis, and eczema (Wan et al. 2024). Additionally, exposure to biocidal agents such as triclosan, parabens, and 3-phenoxybenzoic acid has been positively associated with the development of AD. A study conducted in South Korea found a correlation between urinary levels of triclosan and the prevalence of AD in children and adolescents (Choi et al. 2024).

Genetic mutations plays a central role in AD onset and at least two independent variants from filaggrin encoding gene (*FLG)*, R510X and 2282del4, causes barrier loss of function, representing a very strong predisposing factor for AD (Marenholz et al. 2006). Studies show that loss-of-function mutations in the *FLG* gene are significantly more frequent in patients with moderate-to-severe atopic dermatitis, particularly in European populations, and are associated with more persistent forms of the disease, earlier onset, and an increased risk of atopic comorbidities (Marenholz et al. 2006; Palmer, et al. 2006).

Filaggrin (FLG), one of the main structural proteins of the epidermis, plays a vital role in aggregating keratin intermediate filaments, thereby contributing to the structure and function of the stratum corneum (Steinert et al. 1981). Additionally, filaggrin breakdown products such as urocanic acid and pyrrolidone carboxylic acid assist in skin hydration and maintenance of the acidic pH of the stratum corneum (Jungersted et al. 2010; Miajlovic et al. 2010). Loss-of-function mutations in the FLG gene, which encodes filaggrin, are associated with increased susceptibility to asthma, elevated IgE levels, and particularly with impaired skin barrier function. This leads to increased transepidermal water loss (TEWL) and greater vulnerability to irritants, allergens, and pathogens (Jungersted et al. 2010).

In animal models of atopic dermatitis, early barrier dysfunction leads to skin dryness and increased transepidermal water loss, disrupting cutaneous defenses and triggering innate and adaptive type 2 immune responses (Kubo et al. 2012). These responses are characterized by the presence of T helper 2 (Th2) lymphocytes and the production of cytokines such as thymic stromal lymphopoietin (TSLP), IL-4, IL-5, and IL-13, which promote immune cell recruitment and elevated serum IgE levels across different disease stages (Eb and S. U, 2011).

In early AD, even in non-lesional skin, keratinocytes, macrophages, and other innate immune components are stimulated to produce proinflammatory cytokines such as CCL17, CCL22, IL-1β, IL-25, IL-33, and TSLP. These mediators activate and recruit group 2 innate lymphoid cells (ILC2s) and Th2 lymphocytes (Weidinger and Novak 2016). TSLP also induces expression of the OX40L ligand, which activates naive T cells to secrete IL-4, IL-5, and IL-13. As AD progresses to acute skin lesions, inflammatory responses intensify with antigen presentation by dendritic cells, further activating Th2 cells. IL-4 and IL-13 produced by Th2 cells promote the differentiation of B lymphocytes into IgE-secreting plasma cells. Infiltrating T cells and eosinophils expressing skin-homing adhesion molecules and histamine receptor H4R migrate from the bloodstream to the lesional skin (Weidinger and Novak 2016). In chronic AD, all early immune responses may persist and be accompanied by Th1- and Th17-mediated pathways with variable activation. Moreover, IL-33, TSLP, and Th2 cytokines can interact with peripheral neurons, triggering central sensory responses associated with pruritus (Weidinger and Novak 2016).

### Epidemiology

The epidemiology of AD is uncertain, and studies on its global prevalence and incidence are scarce, especially for the adult population (Laughter et al. 2021). A systematic analysis encompassing 344 studies estimated that approximately 2.6% of the global population is affected by AD, equating to about 204 million individuals (Tian et al. 2023).

A cross-sectional study conducted in 2009 demonstrated that the prevalence of atopic dermatitis (AD) in childhood exhibits a high degree of variability, which can be partially attributed to socioeconomic disparities among nations (Odhiambo et al. 2009). Among children aged 6 to 7 years, the prevalence of AD ranges from 0.9% to 22.5%, whereas in adolescents aged 13 to 14 years, the range is from 0.2% to 24.6% (Odhiambo et al. 2009). Moreover, children with AD are at an increased risk of developing other atopic comorbidities, such as asthma and allergic rhinitis (Johnson et al. 2023).

The prevalence of AD in adults is underdiagnosed, and significant discrepancies are often observed in the available data, the main reasons being the lack of a common diagnostic tool, the challenge of classifying the severity of AD in these patients and the underdiagnosis obtained by different health professionals (Maspero et al. 2023). In an observational cross-sectional study carried out in 2023, the prevalence of AD in adults ranged from 3.4% in Israel to 33.7% in Thailand, with females appearing to be more prone to AD than males (Maspero et al. 2023).

### Clinical features and diagnosis

The most striking feature of AD experienced by patients is intense itching. Unlike common histamine-induced pruritus, in AD, itching originates through a nonhistaminergic pathway resulting from a complex interaction between cutaneous neuronal components, keratinocytes and immune system cells (Biazus Soares et al. 2024).

Histological findings in acute cases of AD reveal intercellular edema of the epidermis (spongiosis) and perivascular infiltration in the dermis of white cells, represented mainly by lymphocytes, mast cells and Langerhans cells, which may also include monocytes, macrophages, neutrophils, eosinophils and basophils (Weidinger et al. 2018).

The chronic inflammatory condition of AD often results in lichenized skin lesions with a pronounced state of acanthosis (thickening of the epidermis), hyperkeratosis, parakeratosis, mononuclear cell infiltration in the dermis and fibrosis (Weidinger et al. 2018). Patients with black or brown skin tones tend to develop post inflammatory hypo or hyperpigmentation in lesions in the presence of lichenified plaques, which has a significant negative impact on their quality of life (Kaufman et al. 2018). At present, there are no objective laboratory tests for diagnosing AD, and the adoption of clinical diagnostic criteria remains the main means used (Weidinger and Novak 2016).

In 1980, Hafkin and Rajka proposed the diagnosis of AD on the basis of the observation of at least three major clinical criteria, such as itching, dermatitis on flexural surfaces and personal or family history of skin or respiratory atopy. Three minor clinical criteria were also described, such as characteristics called"atopic facies”, AD triggers and their complications (Rothe and Grant-Kels 1996).

The work of Hafkin and Rajka served as the basis for the diagnosis of AD and has undergone some modifications over time; currently, the criteria used are based on the parameters adopted by the American Academy of Dermatology (AAD), the joint task force of the American Academy of Allergy, Asthma & Immunology (AAAAI) and the American College of Allergy, Asthma & Immunology (ACAAI) (Feldman, et al. 2019). The diagnosis of atopic dermatitis should be established through a comprehensive assessment of clinical manifestations, with particular emphasis on the presence of chronic, pruritic, eczematous lesions that display characteristic morphology and distribution patterns, particularly in individuals with a personal or family history of atopy. Differential diagnoses should consider other dermatoses with overlapping clinical features, such as seborrheic dermatitis, contact dermatitis, and psoriasis (Schneider et al. 2013).This approach allows the exclusion of other skin conditions with morphology similar to AD (Feldman, et al. 2019).

The manifestations of AD are specific to the acute or chronic stage of the disease and the age of the patient (Clebak et al. 2023). In children, AD is usually acute, with lesions on the face and extension of the limbs; however, from the age of two, the manifestations are polymorphic and diverse and are commonly observed in flexural folds (Weidinger and Novak 2016). In adolescence and adulthood, the lesions present as lignified and excoriated plaques on flexures, wrists, ankles and eyelids, and adults may have chronic eczema and/or pruritic lesions on the palms of their hands (Weidinger and Novak 2016).

## Available therapies

As the pathogenesis of AD is complex and multifactorial, there are many therapeutic approaches available to control and manage the symptoms of the disease, ranging from prophylactic measures to nondrug approaches and the use of drugs from different classes, such as topical and systemic corticosteroids, calcineurin inhibitors, Phosphodiesterase-4 (PDE4) and Janus kinase 4 (JAK4) inhibitors respectively (Eichenfield et al. 2017).

To help make decisions about the management and control of the disease, various guidelines are available to guide and facilitate the approaches that can be adopted by professionals. Among the most recent guidelines are the 2023 AAAAI/ACAAI JTF Atopic Dermatitis Guideline Panel, whose purpose is to provide evidence-based recommendations for the optimal management of AD (Chu et al. 2024).

### Topical treatments

Dry skin (xerosis) is one of the most obvious symptoms of AD, and the use of moisturizers can help restore skin hydration, which is compromised by the loss of barrier function and transepidermal water loss (TEWL) (Nicol et al. 2021). Moisturizers can have emollient properties that promote increased skin lubrication, occlusive properties that prevent water evaporation and humectant properties that increase moisture retention in the skin, counteracting the effects of TEWL often associated with irritated skin in AD (Brooks and Yosipovitch 2024). A wide variety of components can help in the topical treatment of the disease, with moisturizing formulations containing glycerol, colloidal oatmeal, ceramides and even topical products containing probiotics that prevent infection of damaged skin by *Staphylococcus aureus*, a common comorbidity in more severe cases of the disease (Breternitz et al. 2008; Diluvio et al. 2019; Nakatsuji et al. 2016).

The JTF does not recommend the formal prescription of moisturizers because, given the level of evidence available, patients could benefit from more accessible over-the-counter moisturizers, avoiding the high costs and inconveniences normally associated with prescription moisturizers (Chu et al. 2024). A moisturizer should be applied immediately after bathing, all over the body, including areas that appear normal (Saeki et al. 2022). In addition, the continued use of hydration is indicated, even after successful treatments with anti-inflammatories, to maintain remission of the condition (Saeki et al. 2022). When moisturizers alone are insufficient to control AD, additional topical therapeutic strategies become necessary, involving agents from different pharmacological classes (Zuuren et al. 2017; Eichenfield et al. 2017).

The use of TCSs represents one of the fundamental pillars of therapy for AD, and there is a high degree of evidence that this approach is effective in cases where the use of moisturizers as an initial measure fails (Okwundu et al. 2018) (Fishbein et al. 2019). TCSs are usually classified into potency classes, with class VII being considered the mildest (e.g., hydrocortisone 1% or 2.5%) and class I the most potent (e.g., betamethasone dipropionate 0.05%). Several factors determine the potency of a corticoid, such as the intrinsic characteristics and concentrations of the base substances in which it is conveyed (Campos 2018). Systematic reviews have revealed that the use of TCSs for a period of 2–6 weeks probably does not induce the adverse events often associated with continuous therapy with these drugs, such as infections, atrophy, hypertrichosis or local changes in the skin (Axon et al. 2021). It is recommended to avoid the use of high-potency TCSs, specifically class 1 and 2 agents, for durations exceeding four weeks, and to restrict their application on sensitive areas such as the face, intertriginous regions, and the groin due to an increased risk of local adverse effects (Hengge et al. 2006).

Another highly relevant topical therapy for the treatment of AD is the use of calcineurin inhibitors (TCIs), which are cytoplasmic proteins strongly associated with the inflammatory response of lymphocytes and dendritic cells in the skin; these proteins act mainly as transcription factors for IL-2, IL-3, IL-4 and TNF-α (Beltran and Castro 2006).The topical TCIs available and recommended by the JTF guide are pimecrolimus and tacrolimus, whose therapeutic responses are similar to those of class 5 and 6/7 TCSs, respectively (Chu et al. 2024).

Phosphodiesterase-4 (PDE4) is highly expressed in immune cells and is closely related to the production of inflammatory mediators in various skin conditions (Schafer et al. 2014). The inhibition of PDE4 decreases the degradation of cAMP, enabling the suppression of various proinflammatory signaling pathways and decreasing peroxide generation and immune cell chemotaxis (Dong et al. 2016). Although many PDE4 inhibitors are in development, only crisaborole (Eucrisa®—Pfizer) is available in the United States and is recommended for mild to moderate dermatitis in patients two years of age and older (“FDA aprova Eucrisa para eczema | FDA”. 2025). Oral phosphodiesterase-4 (PDE4) inhibitors, such as Orismilast, are currently undergoing clinical investigation for the treatment of atopic dermatitis, however, to date, only topical formulations have been approved and are available for this purpose (Silverberg et al. 2023; Takahashi et al. 2024).

The latest class of topical drugs associated with the treatment of AD are Janus kinase 4 (JAK4) inhibitors. Although many developments are underway, in the United States, only ruxolitinib (Opzelura®—Incyte) is available for the treatment of AD (Chu et al. 2024). Despite the demonstrated efficacy of ruxolitinib in the treatment of atopic dermatitis, its use is currently recommended only for patients aged 12 years and older, due to the frequency and severity of associated adverse events (Papp et al. 2023). JAK4 inhibitors are potent immunosuppressants and can cause serious side effects, such as increased infections, thrombocytopenia and anemia dyslipidemia (Zeiser et al. 2021). Other JAK inhibitors are available for oral administration, such as tofacitinib, a JAK1/2 inhibitor (Chovatiya and Paller 2021).

As a complement to topical drug therapy, treatments such as occlusive applications of bandages moistened with corticosteroids on lesions and the use of topical antimicrobials for infection can be used (Chu et al. 2024).

### Systemic treatments

Topical therapies are the first-line treatment for managing the symptoms of atopic dermatitis. However, moderate to severe cases may not respond adequately to these approaches, requiring systemic treatment, including subcutaneous administration of monoclonal antibodies and oral medications such as cyclosporine, JAK inhibitors, azathioprine, methotrexate and mycophenolate mofetil (Saeki et al. 2022).

Monoclonal antibodies used in systemic treatment via the subcutaneous route include dupilumab (Dupixent®—Sanofi), tralokinumab (Adtralza®—Adbry®—Leo Pharma) and nemolizumab (Nemluvio®—Galderma) (Chang and Nadeau 2017; Wollenberg et al. 2021; Kabashima et al. 2022). These agents act by blocking or modulating the interleukin signalling pathways, which play a key role in inflammatory responses (Nevid and Boguniewicz 2024).

Systemic treatments via oral administration are represented by janus kinase (JAK) inhibitors such as baricitinib (Olumiant®—Eli Lilly), abrocitinib (Cibinqo®—Pfizer) and upadacitinib (Rinvoq®—Abbvie), purine synthesis inhibitors (azathioprine), calcineurin inhibitors (cyclosporine), antimetabolites that interfere with folic acid synthesis (methotrexate) and immunosuppressants such as mycophenolate mofetil, an inhibitor of inosine-5'-monophosphate dehydrogenase also involved in purine synthesis (Lebwohl et al. 2022).

Activation of immune cells requires intense cellular proliferation, which is directly dependent on the availability of purine nucleotides, such as ATP and GTP (Cekic and Linden 2016). Purine synthesis inhibitors, such as azathioprine and mycophenolate mofetil, reduce this proliferative capacity, thereby attenuating the inflammatory response (Meggitt et al. 2006; Allison and Eugui 2000).

Although systemic therapy for severe and refractory conditions represents the last line of therapy, the drugs associated with this approach are usually accompanied by more severe adverse events and require a detailed clinical history of the patient (Zhang et al. 2025; Viermyr et al. 2025; Snijders et al. 2024). In addition, some therapies have high costs, such as the use of monoclonal antibodies, leading to an increase in health judicialization.

## Cutaneous endocannabinoid system

The endocannabinoid system is composed mainly of canonical CB1/CB2 receptors and various endocannabinoid substances, with anandamide (AEA) and 2-arachidonoyl-glycerol (2-AG) being the main agonists involved (Marzo et al. 2004). Enzymes such as fatty acid amide hydrolase (FAAH) and monoacylglycerol lipase (MAGL) are key modulators of the endocannabinoid system. FAAH limits cannabinoid signaling by degrading AEA and palmitoylethanolamide (PEA), while MAGL hydrolyzes 2-AG thereby regulating the intensity and duration of endocannabinoid activity (Goparaju et al. 1998; Dinh et al. 2004). In addition to the receptors characteristic of the endocannabinoid system, other receptors, such as ion channels activated by transient receptor potential (TRP), can be modulated by cannabinoids (Kim et al. 2015). CB1 and CB2 are protein-coupled receptors (GPCRs) that act to regulate the concentrations of intracellular second messengers, such as adenylate cyclase (AC), mitogen-activated protein kinase (MAPK) and calcium ions (Ca^2+^) (Marzo et al. 2004). CB1 is widely distributed in the central nervous system and, to a lesser extent, in the peripheral nervous system (Han et al. 2023; Agarwal, et al. 2007).

The evidence of the presence of CB1 and CB2 receptors is numerous and has accompanied the discovery of the endocannabinoid system since the 1980 s (Devane et al. 1988; Munro et al. 1993). However, recent studies have shown the presence of these receptors in tissues and systems that have not been previously studied, such as the skin and skin barriers (Ständer et al. 2005). A study involving immunofluorescence carried out in 2005 revealed for the first time the presence of CB1 receptors in keratinocytes in the stratum corneum and granulosum of human skin and CB2 receptors in basal keratinocytes, as well as the presence of these receptors in nerve fibers in the epidermis, mast cells, macrophages and skin appendages such as follicles and glands (Ständer et al. 2005).

Developments in the use of cannabinoid agonists and antagonists of the endocannabinoid system in the skin include potential therapeutic targets for treating conditions such as abnormal cell proliferation, acne, baldness, skin cancer and inflammation (Sheriff et al. 2020). In the context of AD, cannabinoids can be explored for the use as a topical, subcutaneous or oral administration route as reported by some experimental studies, mainly in animal models (Gaffal et al. 2013; Karsak et al. 2007). Although no clinical and pre-clinical reports are currently available, case reports series with humans and experimental studies with mice suggest that oral administration of cannabinoids may modulate peripheral nerve transmition involved with itch (Morin et al. 2021; Lou et al. 2022; Karsak et al. 2007).

Cannabinoids, whether of endogenous, plant or synthetic origin, have demonstrated anti-inflammatory and antipruritic properties in diseases such as atopic dermatitis, contact dermatitis and psoriasis, with mechanisms involving both the regulation of the endocannabinoid system and the modulation of immune responses present in these conditions (Sheriff et al. 2020).

## Cannabinoids as complementary therapy for atopic dermatitis

Pruritus, or itching, is one of the most obvious symptoms of atopic dermatitis and has a complex pathophysiological mechanism that involves concomitant neurological and immunological responses and stimuli. The act of scratching begins with the transmission of stimuli captured in the skin by peripheral neural fibers to the dorsal root and trigeminal ganglion, which stimulate and activate the spinal cord and brain to trigger behaviors related to itching (Li et al. 2024). At least six receptors are currently known to be involved in the mechanism of pruritus, which can be divided into histaminergic and nonhistaminergic pathways (Guo et al. 2022).

The histaminergic pathway is usually the most relevant pathway in studies involving pruritus and is involved in mainly type 1 hypersensitivity reactions that occur in response to intracellular pathogens such as viruses (Weidinger and Novak 2016). In this pathway, histamine is released by the degranulation of mast cells and basophils, activating sensory neurons that express H1R and H4R receptors, as well as the TRPV1 transient ion channel (Bautista et al. 2014).

Despite the involvement of the histaminergic pathway in triggering pruritus, its relevance in the context of atopic dermatitis remains in the background since treatment with antihistamines is refractory (Weidinger and Novak 2016; Guo et al. 2022). The expression of nonhistaminergic pruritogens such as endothelin I (ET1), which activates TRPV and TSLP receptors, and type 2 cytokines such as IL-4, IL-13 and IL-31 seems to be more relevant in this context, and the use of drugs such as oral and topical Janus kinase (JAK) inhibitors, which are common mediators in some of these pathways, is more efficient (Weidinger and Novak 2016).

Transient receptor potential (TRP) receptors are ion channels expressed in different human tissues and play crucial roles in cell signaling via various sensory mechanisms (Rahman et al. 2024). TRP receptors are classified into several subfamilies, with vanilloid TRP (TRPV) comprising six representatives (TRPV1-6), which have convergent mediators in processes such as hyperalgesia, pain and itching (Rahman et al. 2024; Yosipovitch et al. 2024). The TRPV1 channel (capsaicin receptor) can be modulated by cannabinoids, preventing neuronal activation of inflammatory mechanisms associated with itching (Smart et al. 2000; Singto et al. 2025). PEA, an endocannabinoid analog of AEA, is one of the most studied TRPV1 agonists for reducing itching (Ambrosino et al. 2013). Although PEA has low affinity for the canonical CB1 and CB2 receptors, it appears to exert antipruritic effects indirectly via CB2 receptor modulation, primarily through its interaction with TRPV1 channels. This mechanism is linked to the inhibition of AEA degradation, representing a classic example of the'entourage effect'within the endocannabinoid system (Eberlein et al. 2008; Lo Verme, et al. 2005). TRPV1 channels act as co-receptors of CB2 in keratinocytes, and PEA-mediated modulation of these channels leads to the inhibition of FAAH, the enzyme responsible for AEA breakdown (Lastname 2018).

The phytocannabinoids cannabidiol (CBD) and Δ−9-tetrahydrocannabinol (THC) are also compounds that need to be explored in terms of itch control. An observational study of patients with epidermolysis bullosa revealed that the use of cannabinoids to control itching and pain was mainly topical with formulations containing combinations of CBD/THC and CBD alone, with approximately 90.9% of patients reporting significant improvement with CBD-containing formulations (Schräder et al. 2021). In another study, the application of a topical formulation based on CBD and aspartame for 14 days in patients diagnosed with atopic dermatitis significantly improved symptoms compared with those in the placebo group, although the use of a formulation containing only CBD did not yield significant results (Gao et al. 2022). Although the potential of phytocannabinoids as antipruritus agents is promising, especially in relation to CBD, the results concerning their efficacy often differ across studies involving animals and humans, and further research is needed (Loewinger et al. 2022; Mariga et al. 2023; Gao et al. 2022).

While the majority of studies advocate for the topical administration of cannabinoids, oral delivery of these compounds also represents a viable approach that warrants further investigation for the alleviation of AD. At a central level, CB1 stimulation can result in a reduction in both inflammation and itching. Case reports of patients with neuropathic itching indicate that after oral use of dronabinol (Syndros®—Benuvia Operations), a synthetic analog of Δ−9-tetrahydrocannabinol, there was a significant improvement in the degree of visual severity of the lesions (Morin et al. 2021).

Ceramides are important components of the skin that play a role in skin homeostasis, mainly related to protection against foreign agents and reducing TEWL (Sho et al. 2022). Ceramide synthesis is the result of the conjugation of fatty acids and sphingolipids in the stratum basale, followed by glycosylation or conversion into sphingomyelin in the Golgi complex, where it is packaged into vesicles (Meckfessel and Brandt 2014).

As keratinocytes undergo terminal differentiation, particularly in the suprabasal layers such as the stratum spinosum, there is a marked secretion of lamellar bodies containing glycosylated ceramides and sphingomyelin into the extracellular space. These precursors are subsequently hydrolysed by β-glucocerebrosidase and sphingomyelinase, generating free ceramides. Together with cholesterol and free fatty acids, these lipids self-assemble into lamellar structures in the stratum corneum, forming the basis of the epidermal barrier (Schräder et al. 2021).

Patients with atopic dermatitis usually have reduced levels of ceramide in the stratum corneum as well as differences in the functional profile of ceramides present, which has a significant effect on the loss of skin barrier function, especially related to TEWL (Janssens et al. 2012). A study involving keratinocyte cultures from patients with psoriasis revealed that treating cells with 4 µM cannabidiol (CBD) stimulated sphingomyelinase activity with a consequent increase in ceramide levels, which may contribute to maintaining the structure of the epidermis and preventing water loss (Łuczaj et al. 2020; Meckfessel and Brandt 2014).

Aquaporins (AQPs) are key proteins for skin physiology and homeostasis and are responsible for the transport of water and neutral solutes in cell membranes, which has direct implications for the hydration status of the skin (Silva et al. 2021). The expression of AQP3, an isoform present considerably in keratinocytes, can be modulated by the application of a 1% CBD solution for 14 days in a hairless mouse model (Ikarashi et al. 2021). Compared with those in the control group, the water content in the skin of CBD-treated animals increased significantly, and the expression levels of mRNAs related to AQP3 increased proportionally in the groups exposed to CBD (Ikarashi et al. 2021).

The pronounced expression of the CB1 and CB2 receptors in immune cells and lymphoid tissues involved in innate and adaptive immune responses makes the use of cannabidiol a potential mediator of inflammatory responses (Ruhl et al. 2021; Laprairie et al. 2015) I. In a study involving knockout animals, the absence of the CB1 receptor increased IL-4, TSLP and CCL-8 levels as well as increased neutrophil activity, in addition to delaying epithelial barrier repair, as observed in AD patients (Gaffal et al. 2014). CBD is known to decrease the expression of proinflammatory factors such as NF-κ B and TNFα, which consequently can reduce the inflammatory activity of various pathways that are based on the signaling of these mediators, including interleukins such as IL-6, IL-1,14, IL-5, IL-13, IL-8 and IL-1β, some of which are related to the immunological course of atopic dermatitis (Weidinger et al. 2018; Gęgotek et al. 2021).

Nevertheless, in the context of controlling inflammation, the topical application of THC in a study on contact dermatitis carried out with mice revealed that the activity of this phytocannabinoid contributed to a reduction in inflammation in both the CB1 and CB2 receptor knockout animals and the wild-type group, with a significant reduction in the infiltration of immune cells in the tissues evaluated; the inhibition of IFN-ɣ by T lymphocytes; and a reduction in the production of CCL2, CCL8 and CXL10 by epidermal keratinocytes (Gaffal et al. 2013).

Oxidative stress plays a significant role in the pathophysiology of AD and is characterized by an imbalance between the excessive production of reactive oxygen species (ROS) and the skin’s antioxidant defense mechanisms, leading to cellular damage and chronic inflammation (Ji and Li 2016). Recent studies have demonstrated a significant association between TRPV3 receptor activity and oxidative stress mechanisms involved in the pathophysiology of AD (Song et al. 2024). In canine models, the presence of TUNEL-positive cells, a technique used to identify apoptotic activity, increases dramatically during the spontaneous onset of AD, whereas after treatment with canine adipose tissue stem cell (cATSC) extract, rich on immunomodulatory cytokines, the number of TUNEL-positive cells markedly decreases, partly through the inactivation of oxidative stress-related pathways (Jee et al. 2013). A study on oxidative stress in keratinocytes from patients with psoriasis revealed that CBD promoted an increase in antioxidant properties on cell membranes, preventing lipid oxidation and TWEL (Łuczaj et al. 2020). Other studies also suggest that CBD acts to reduce the formation of reactive oxygen species (ROS) by increasing the activity of endogenous antioxidant mechanisms such as NADPH oxidase, xanthine oxidase and glutathione reductase (Atalay et al. 2021; Wójcik et al. 2020).

In addition to the therapeutic potential mentioned above, especially in relation to regulatory mechanisms inherently linked to the natural course of AD, cannabinoids may also have antimicrobial activity against various bacterial species, including *Staphylococcus aureus*, a microorganism closely related to severe cases of AD (Sionov and Steinberg 2022). The skin microbiota is vital for maintaining homeostasis and immune regulation (Sanchez-Lopez et al. 2025). In atopic dermatitis (AD) the presence of dysbiosis process is marked by an increased presence of *Staphylococcus*, especially *S. aureus*, in contrast with healthy skin, that shows a diverse range of commensal microorganisms such as *Paracoccus sp., Pseudomonas sp., Lactobacillus iners, Streptococcus thermophilus* and other bacteria species (Gomes et al. 2024).

A study on the stability of three oily formulations containing CBD and THC extracted from *Cannabis sativa* demonstrated antimicrobial activity against *Escherichia coli*, *S. aureus*, and *Paenibacillus larvae* (Werner et al. 2024). In addition to CBD and THC, other studies have reported that cannabichromene (CBC), cannabigerol (CBG), and cannabinol (CBN) exhibit potent activity against various strains of methicillin-resistant *S. aureus* (MRSA) (Gęgotek et al. 2023). Although the exact antibacterial mechanism of action of cannabinoids remains unclear, it has been hypothesized that they may disrupt the integrity of the bacterial cytoplasmic membrane (Werner et al. 2024) Fig. 1.

**Fig. 1:**
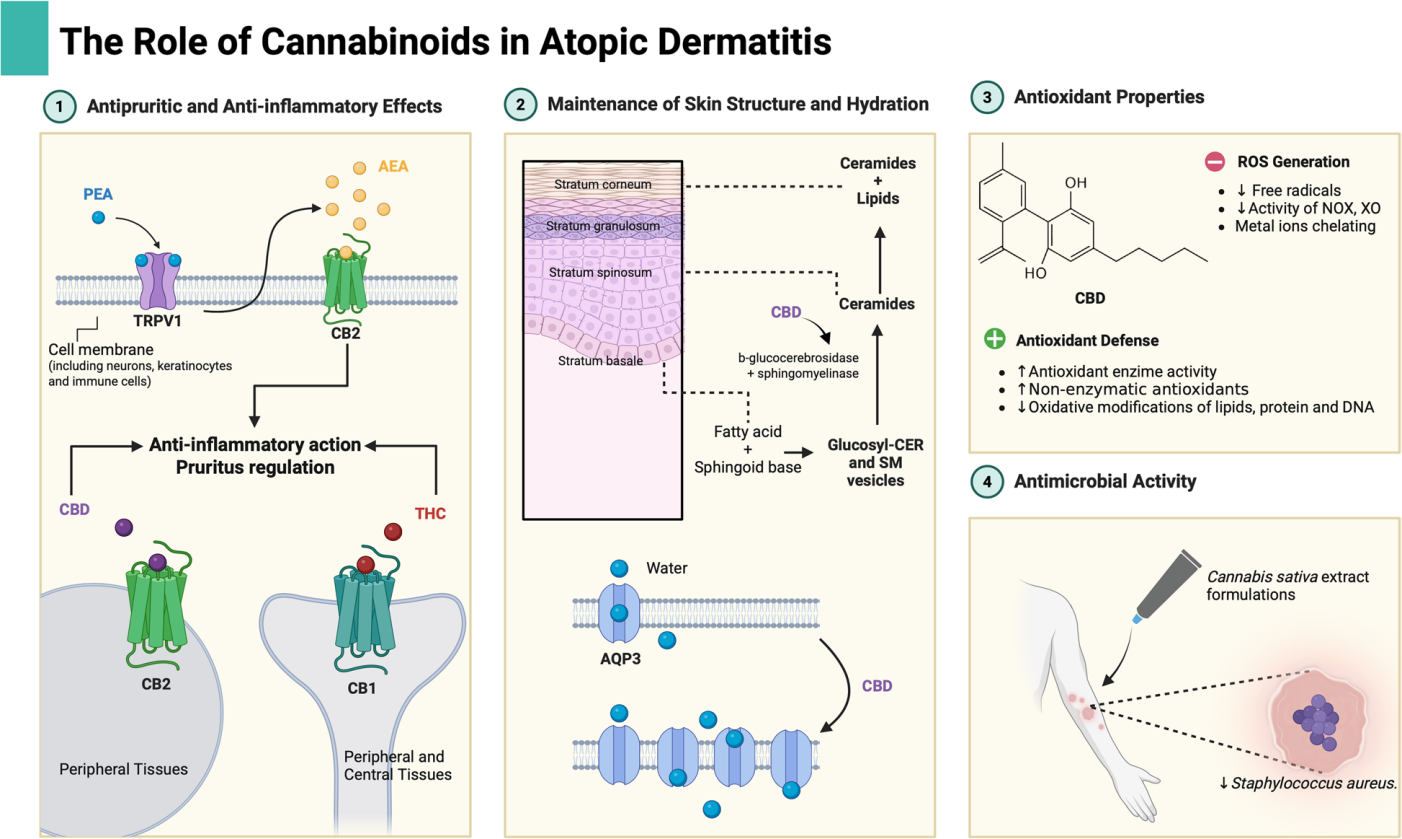
Cannabinoids as complementary therapy for DA. 1 PEA can interact with TRPV1 and prevent degradation of AEA, an anti-inflammatory and antipruritic endocannabinoid; Phytocannabinoids such CBD and THC interact with CB receptors and also have anti-inflammatory effects. 2 CBD increase sphyngomielinase activity and expression of AQP3, contributing to the maintenance of skin structure and hydration, respectively. 3 CBD possesses antioxidant properties, exerting both direct and indirect effects on the reduction of free radical formation and on protective mechanisms. 4 Formulations containing CBD and THC derived from *Cannabis sativa* extract have antimicrobial activity against *S. aureus* and other microorganisms. *Created in Biorender. Geraldo, A. (2025) *https://Biorender.com/pfekfox

## Final considerations

Cannabinoids, whether of plant, endogenous, or synthetic origin, clearly possess significant therapeutic potential and should be further explored as complementary treatments for AD. The development of cannabinoid-based formulations for skin conditions is not limited to products classified as medicines by pharmaceutical regulatory agencies, but also includes their use as active ingredients in cosmetic formulations, such as soaps, shampoos, and especially moisturizing lotions and creams, for individuals with AD and other conditions requiring enhanced skin hydration.

Beyond the therapeutical potential of the classical phytocannabinoids CBD and THC, other components such as CBG and CBC have also been investigated for their dermatological benefits, including anti-inflammatory, antibacterial, and antioxidant properties that may contribute to skin health and the treatment of various skin disorders, including AD (Gęgotek et al. 2023; Long et al. 2025; Chaiwangrach et al. 2024).

Although the number of studies involving cannabinoids has increased in recent years, further research is still necessary, particularly regarding the safety of these compounds for human use and their efficacy in inducing remission of AD, as current findings often present contradictory results.

Regarding the phytocannabinoids, the development of *Cannabis sativa* derived products not only represents a clear advancement in the innovation, production, and commercialization of high-value technologies such as pharmaceuticals and cosmetics, but also holds the potential to generate significant benefits for the national agricultural sector of the countries (Schluttenhofer and Yuan 2017). Agronomically, *cannabis* species are characterized by rapid growth, high productivity, and adaptability to various soil and climatic conditions, making it attractive for diverse farming regions (Andre et al. 2016). Is important to underscore the urgent need for the harmonization and liberalization of regulatory frameworks regarding *Cannabis sativa* among the countries, in order to facilitate the continued advancement of scientific research, industrial production, and the therapeutic use of cannabinoid-based products for medical purposes.

## Data Availability

No datasets were generated or analysed during the current study.

## Abbreviations

AAAAI: American Academy of Allergy, Asthma & Immunology
ACAAI: College of Allergy, Asthma & Immunology
AD: Atopic Dermatitis
CBC: Cannabichromene
CBD: Cannabidiol
CBG: Cannabigerol
CBN: Cannabinol
FLG: Filaggrin
JAK: Janus Kinase
JTF: Joint Task Force
PEA: Palmitoyletanolamide
PDE4: Phosphodiesterase-4
TCSs: Topical corticosteroids
TCIs: Topical calcineurin inhibitors
TSLP: Thymic stromal lymphopoietin
TEWL: Transepidermal Water Loss
THC: Δ-9-Tetrahydrocannabinol
